# Rassismus und Diskriminierung im Kontext gesundheitlicher Ungleichheit – ein narratives Review

**DOI:** 10.1007/s00103-023-03764-7

**Published:** 2023-09-21

**Authors:** Katja Kajikhina, Carmen Koschollek, Kayvan Bozorgmehr, Navina Sarma, Claudia Hövener

**Affiliations:** 1 Soziale Determinanten der Gesundheit (Fachgebiet 28), Abteilung für Epidemiologie und Gesundheitsmonitoring, Robert Koch-Institut, General-Pape-Str. 62-66, 12101 Berlin, Deutschland; 2 ÖGD-Kontaktstelle: Infektionsepidemiologisches Krisenmanagement, Ausbruchsuntersuchungen und Trainingsprogramme (Fachgebiet 31), Abteilung für Infektionsepidemiologie, Robert Koch-Institut, Seestraße 10, 13353 Berlin, Deutschland; 3 AG Bevölkerungsmedizin und Versorgungsforschung, School of Public Health, Universität Bielefeld, Bielefeld, Deutschland; 4 AG Epidemiologie & International Public Health, Fakultät für Gesundheitswissenschaften, Universität Bielefeld, Bielefeld, Deutschland; 5 Sektion Health Equity Studies & Migration, Universitätsklinikum Heidelberg, Heidelberg, Deutschland

**Keywords:** Soziale Determinanten der Gesundheit, Teilhabe, Gesundheitliche Gerechtigkeit, Strukturelle Benachteiligung, Migration, Social determinants of health, Social participation, Health equity, Structural discrimination, Migration

## Abstract

Rassismus und Diskriminierung als soziale Determinanten der Gesundheit stehen auch in Deutschland zunehmend im Fokus der Public-Health-Forschung. Studien zeigen Zusammenhänge mit physischer und psychischer Gesundheit bis hin zu Veränderungen auf zellulärer Ebene auf. Neben den gesundheitsschädigenden Effekten interpersoneller und direkter Diskriminierung ist die Relevanz des strukturellen und institutionellen Rassismus für die gesundheitliche Ungleichheit bislang nur wenig beleuchtet. Im Rahmen einer narrativen Übersichtsarbeit werden relevante und aktuelle Forschungsergebnisse zusammengestellt und kritisch diskutiert sowie Handlungsempfehlungen für Forschung und Praxis abgeleitet.

Strukturelle und institutionelle Aspekte von Diskriminierung und Rassismus stehen in engem Zusammenhang mit der gesundheitlichen Lage. So steht die systematische Benachteiligung in den Bereichen Bildung, Arbeit, Wohnen sowie Gesundheitsversorgung im Zusammenhang mit der allgemeinen, psychischen und physischen Gesundheit, mit der Inanspruchnahme von Präventions- und Versorgungsleistungen sowie mit dem Gesundheitsverhalten.

Eine Analyse der Verschränkung von Lebens‑, Wohn- und Arbeitsbedingungen mit der gesundheitlichen Lage von Menschen mit (und ohne) Migrationsgeschichte – generell und in Verbindung mit Rassismus und Diskriminierung – erscheint notwendig, um gezielte Maßnahmen im Hinblick auf Verhältnisprävention abzuleiten, statt auf bloße Verhaltensprävention zu fokussieren. Neben praktischen Interventionen (Trainings, Aufklärungsarbeit, communitybasierten Ansätzen) ist die Weiterentwicklung methodischer Aspekte im Bereich der Erhebung und Analyse von Daten wichtig, um dieser Problemlage umfassend in Forschung und Praxis zu begegnen.

## Abstract (en)

Racism and discrimination as social determinants of health are becoming increasingly recognised in public health research in Germany. Studies show correlations with physical and mental health and even changes at the cellular level. In addition to the adverse health effects of interpersonal and direct discrimination, the relevance of structural and institutional racism for health inequalities has been little explored. This narrative review synthesises and critically discusses relevant and recent research findings and makes recommendations for action in research and practice.

Structural and institutional aspects of discrimination and racism are closely linked to health. Systemic discrimination in education, employment, housing and healthcare affects overall, mental and physical health, access to prevention and care, and health behaviour.

An analysis of the relationship between living, housing and working conditions and the health situation of people with (and without) a history of migration – in general and in relation to racism and discrimination – seems necessary in order to derive targeted measures for structural prevention, rather than focusing on purely behavioural prevention. In addition to practical interventions (trainings, education, and community-based approaches), the further development of methodological aspects in the field of data collection and analysis is important in order to address this issue comprehensively in research and practice.

## Hintergrund

Gesundheitliche Ungleichheit wird maßgeblich durch soziale Determinanten der Gesundheit bestimmt – also durch die Bedingungen, unter denen Menschen aufwachsen, leben, lernen, arbeiten und altern [1]. Politische, gesetzliche, institutionelle und ökonomische Rahmenbedingungen entscheiden als strukturelle Determinanten über die gesellschaftliche Verteilung von Macht und Ressourcen und damit über die sozialen Voraussetzungen einer guten Gesundheit [2]. Weitere Faktoren sind soziale Normen und alltägliche Erfahrungen von Ausschluss und Zugehörigkeit. Diese beeinflussen – in Verknüpfung mit strukturellen Determinanten – die Gesundheit und gesundheitliche Ungleichheit von Menschen weltweit.

Diskriminierung und Rassismus rücken zunehmend in den Fokus der Public-Health-Forschung, zahlreiche Studien zeigen den Zusammenhang mit der Gesundheit der Betroffenen auf [3–8]. Diese nehmen die allgemeine, subjektive und psychische Gesundheit, Zugang zu Versorgung sowie das Gesundheitsverhalten in den Blick [9, 10]. Die Erkenntnisse stammen größtenteils aus dem angloamerikanischen Sprachraum und liefern eine solide Grundlage für eine Diskussion über gesundheitliche Folgen struktureller Ausschlüsse im Kontext von Diskriminierung und Rassismus. Deutschland blickt auf eine lange Geschichte der Migration zurück und bedarf daher einer eigenen Debatte unter Berücksichtigung der hiesigen gesellschaftlichen Entwicklung. Es lohnt daher eine kurze Rückschau auf die Geschichte der Migrationsgesellschaft und die Konjunkturen des Rassismus in Deutschland – mit Blick auf gesundheitliche Folgen.

### Rassismus und Migrationsgesellschaft in Deutschland

Nach der Ermordung von George Floyd 2020 in den USA – im ersten Jahr der COVID-19-Pandemie – verbreitete sich der Slogan „Racism is a pandemic too“ weltweit [11]. In Deutschland wurde er ebenfalls aus aktuellen Gründen aufgegriffen, hier waren im selben Jahr in Hanau 9 junge Menschen aus rassistischen Motiven ermordet worden und im Jahr zuvor hatte es den antisemitischen Anschlag auf die Synagoge in Halle und den rechtsterroristischen Mord an Walter Lübcke gegeben.

Die Debatten waren allerdings in Deutschland nicht neu: Bereits seit 2013 fanden die Gerichtsprozesse im Fall der Mordserie der rechtsextremen Terrorgruppe „Nationalsozialistischer Untergrund (NSU)“ statt. Weitere Beispiele aus einer Reihe von Ereignissen im Kontext rassistischer Gewalt: die rassistischen Pogrome in den 1990er-Jahren in Mölln, Rostock-Lichtenhagen, Solingen und Hoyerswerda [12] und die wiederkehrende und endemische Gewalt gegenüber Geflüchteten vor und nach 2015 [13].

Die Konjunkturen des direkten, strukturellen und institutionellen Rassismus in Deutschland und weltweit stehen in einem unmittelbaren Zusammenhang mit der gesundheitlichen Lage der Betroffenen. Obgleich beispielsweise Migrant:innen häufig eine überproportional gute Gesundheit aufweisen, verschlechtert sich während und nach der Migration in den Transit- und Ankunftsländern ihre Gesundheit. Das Vorkommen chronischer, nichtübertragbarer Erkrankungen konvergiert meist mit deren Häufigkeit in den Zielländern, wohingegen sich die psychische Gesundheit oft verschlechtert ([14], s. auch Beitrag Bartig et al. in dieser Themenausgabe). Mögliche Erklärungen sind Versorgungsbarrieren sowie institutionelle und strukturelle Diskriminierung. Diese werden im Folgenden skizziert.

### Direkte, strukturelle und institutionelle Formen von Diskriminierung und Rassismus

Diskriminierung bedeutet Ungleichbehandlung, Schlechterstellung und Benachteiligung aufgrund von Eigenschaften, die Menschen oder Gruppen zugeschrieben werden. Diskriminierung kann intentional, zielgerichtet, aber auch unbeabsichtigt stattfinden. Sie kann durch einzelne Personen (*direkt, interpersonell*) oder durch Strukturen oder unterschwellige Mechanismen verursacht sein, kann sichtbar oder verdeckt und von den Betroffenen unbemerkt ablaufen – relevant ist der für die Betroffenen entstehende Schaden [15, 16].

*Rassismus* beschreibt nicht lediglich Diskriminierung aufgrund von (vermeintlichen) Merkmalszuschreibungen wie Ethnizität oder Herkunft. Er ist ein komplexes soziales Phänomen, ein Bedeutungssystem, das aus einer Vielzahl von Diskursen, Zuschreibungen und Handlungspraxen besteht. Dabei sind unterschiedliche Ebenen wie politische, nationale, soziale, ökonomische, institutionelle, religiöse oder morphologische eng miteinander verwoben, die zu einer spezifischen Ausdifferenzierung sozialer Beziehungen und gesellschaftlicher Dynamiken von Zugehörigkeit, Ausschlüssen, Wissensproduktion und Arbeitsteilungen führen [17, 18]. Dabei wurden in der Rassenideologie – also der Lehre, dass Menschen in „Rassen“ unterscheidbar sind – zur Begründung der sozialen Konstruktion von „Rasse“ immer biologisch-genetische, morphologische, biologistische und kulturelle Kategorien zur Argumentation herangezogen, wobei die historische Entwicklung zu einer Verlagerung von biologischen hin zu kulturellen Differenzziehungen führte (s. auch: „Rassismus ohne Rassen“ [18, 19]). Diese wurden zu einer Naturalisierungs- und Plausibilisierungsgrundlage für gesellschaftliche Ungleichheiten, Unterdrückungs‑, Ausbeutungs- und Gewaltverhältnisse in Form von Sklaverei, Kolonialismus, Verbrechenskomplexen des Nationalsozialismus und der Eugenik – bis hin zu aktuellen Ausschlüssen im Zugang zu gesellschaftlichen Ressourcen wie Bildung, Arbeit, Einkommen, Wohnen und Gesundheitsversorgung (soziale Determinanten der Gesundheit), auf die hier beispielhaft eingegangen werden soll [20, 21].

Unterschiedliche Teilaspekte von Rassismus als Praxis auf alltäglicher sowie institutioneller und struktureller Ebene sind beschrieben. „*Othering*“ („Ver-Anderung“) ist ein Konzept, das ursprünglich aus postkolonialer Theorie stammt und inzwischen auch Einzug in die Public-Health-Debatte gehalten hat. Es beschreibt die (Re)Produktion von Stereotypen, Vorurteilen, stigmatisierenden Bildern und Kategorien sowie die Abgrenzung zwischen einem „Wir“ und den „Anderen“, oft einhergehend mit einer Abwertung und Zuschreibung von Eigenschaften und Verhaltensweisen ([22–24], s. auch Beitrag von Akbulut et al. in dieser Themenausgabe). Auch die Praktiken des (Ver)Schweigens und Ignorierens von bestimmten Bevölkerungsgruppen oder Themenbereichen (engl.: „*omission*“) werden als Dimensionen von Rassismus diskutiert – wie etwa die Abwesenheit bestimmter Themen in Ausbildungsinhalten oder auch die systematische Unterrepräsentation in bevölkerungsbezogenen Daten und im Berichtswesen [25, 26].

Unter *strukturellem Rassismus* wird das Zusammenspiel gesellschaftlicher Bedingungen auf der Makroebene verstanden, welches die Chancen, Ressourcen und das Wohlergehen von Personengruppen aufgrund ihrer (vermeintlichen) Ethnizität, Herkunft, Community-Zugehörigkeit, ihres Aussehens, Namens oder anderer Rassifizierungsmerkmale einschränkt und zugleich anderen – *weißen*^1^ – Personengruppen Macht, Zugang zu Ressourcen und Privilegien ermöglicht und sichert. Struktureller Rassismus zeigt sich in eingeschränkter Teilhabe und einem begrenzten Zugang zu gesellschaftlichen Ressourcen, einschließlich Gesundheit, Justiz, Bildung, Wohnraum und Arbeitsmarkt für Menschen, die in Verbindung mit Rassifizierung mit Abwertungsprozessen und damit verbundenen systematischen und immer wiederkehrenden Ausschlüssen konfrontiert sind [27, 28]. Struktureller Rassismus kann in Verbindung mit weiteren Status- und Ungleichheitsmerkmalen wie Geschlechtsidentität, sexueller Orientierung, chronischen Einschränkungen, Klasse oder sozioökonomischem Status, Community-Zugehörigkeit oder Kenntnissen der Mehrheitssprache auch zu multipler Benachteiligung führen [28]. Bei der Analyse dieser Verschränkungen, der Mehrfachdiskriminierung sowie der daraus resultierenden Erfahrungen und gesellschaftlichen Positionen ist das von Schwarzen Feministinnen entwickelte Konzept der Intersektionalität hilfreich und unumgänglich [29–31].

*Institutioneller Rassismus* bezeichnet hingegen die Einbettung und Sicherung von Ausschlüssen von gesellschaftlicher Teilhabe, aber auch von rassistischen Wissensbeständen in Form von politischen, legislativen und institutionellen Strukturen, Gesetzgebungen und Praktiken. Damit geht auch eine systematisch geringere Beteiligung bestimmter Bevölkerungsgruppen an gesellschaftlichen Gestaltungsmöglichkeiten einher [32, 33]. Einige Diskriminierungsformen sind nach europäischen und landesspezifischen gesetzlichen Richtlinien verboten [34]. Andere Formen wiederum sind sogar gesetzlich angeordnet.^2^

Ziel dieses Beitrags ist es, ohne Anspruch auf Vollständigkeit, einen Überblick zu *strukturellen* und *institutionellen* Aspekten von Rassismus und zu deren direkten sowie indirekten Verschränkungen mit gesundheitlicher Ungleichheit zu geben. Zudem werden die gesundheitsschädigenden Auswirkungen direkter interpersoneller rassistischer Diskriminierung (z. B. Hassverbrechen, verbale oder körperliche Gewalt, schlechtere Behandlung) oder Internalisierung rassistischer Abwertung (z. B. Selbstabwertung, Hoffnungslosigkeit) thematisiert.

Hierzu wurde eine umfassende, jedoch nicht systematische Literaturrecherche durchgeführt. Weiterhin sind die Ergebnisse eines Scoping-Reviews zum Thema „Operationalisierung und Erhebung von (Anti‑)Diskriminierungsdaten in der Gesundheitsforschung“ eingeflossen, das im Rahmen des Projekts IMIRA (Improving Health Monitoring in Migrant Populations) durchgeführt wurde (nicht publiziert).

## Struktureller Rassismus, Diskriminierung und Gesundheit

Diskriminierung, Ungleichbehandlung und gesundheitliche Ungleichheit treten in einer krisenhaften Lage wie der COVID-19-Pandemie verstärkt zutage. Studien aus den Vereinigten Staaten und dem Vereinigten Königreich zeigten eine höhere Betroffenheit von afroamerikanischen, Schwarzen und anderen rassifizierten Communitys im Hinblick auf die COVID-19-Inzidenz und Mortalität [36, 37]. Einige lokale Datenerhebungen und erste ökologische Analysen bestätigten dies auch für Deutschland [38–40]. Hinweise auf Korrelationen mit der Arbeits- und Wohnsituation, aber auch Indikatoren wie der Deprivationsgrad der Wohnumgebung liefern Erklärungsmuster für diese Beobachtungen [41, 42]. Es gibt Hinweise darauf, dass Diskriminierung eine wichtige Rolle bei diesen Unterschieden spielt [43]. Auch jenseits der Pandemie zeichnen sich deutliche gesundheitliche Unterschiede für rassifizierte und migrantische Communitys^3^ ab. So wird eine höhere Krankheitslast durch nichtübertragbare Erkrankungen wie Diabetes Typ II, Bluthochdruck, Herz-Kreislauf-Erkrankungen, Krebs sowie psychische Erkrankungen für afroamerikanische Communitys in den USA, aber auch für migrantische Communitys in Europa festgestellt [45, 46].

### Zusammenhänge zwischen sozialer, struktureller und gesundheitlicher Ungleichheit

Die Verknüpfung zwischen sozialer Ungleichheit, gesundheitlichen Risiken und erhöhter Krankheitslast hinsichtlich übertragbarer und nichtübertragbarer Erkrankungen wird über mehrere Wege vermittelt. Psychosoziale Folgen für die Gesundheit entstehen aufgrund von traumatischen Erfahrungen einschließlich institutioneller und interpersoneller Diskriminierung und Gewalt. Zugleich spielen ökonomische und soziale Deprivation, Mangel und Unsicherheit in Bezug auf (gesunde) Ernährung und gesunde Lebenswelten mit Möglichkeiten physischer und psychischer Erholung eine Rolle. Belastungen und Expositionen gegenüber toxischen, infektiösen oder schädlichen Substanzen bei der Arbeit und in der Wohnumgebung tragen zu einem erhöhten Risiko für dauerhafte gesundheitliche Beeinträchtigungen bei. Auch die unzureichende Gesundheitsversorgung aufgrund gesetzlicher und versicherungsbezogener Einschränkungen sowie direkter Diskriminierung im Gesundheitswesen ist mit gesundheitlicher Ungleichheit assoziiert [47, 48].

Gesundheitliche Ungleichheit wird aber auch über Aktivitäten des privaten Sektors vermittelt, wie etwa durch zielgruppenspezifisches Werbetargeting von ungesunden Produkten wie Fastfood, Softdrinks, Alkohol, Tabak oder psychoaktiven Substanzen (*„commercial determinants of health“*; [49, 50]). Dieser Aspekt ist allgemein gesundheitsrelevant, jedoch als besonders schwerwiegend für benachteiligte Bevölkerungsgruppen zu betrachten: Von Diskriminierung und dauerhafter sozioökonomischer Benachteiligung betroffene Personen zeigen ein höheres Risiko für gesundheitsschädigendes Verhalten im Bereich Ernährung und Substanzmissbrauch (beschrieben als „*emotion-focused strategies*“, eine der möglichen Bewältigungsstrategien von Diskriminierung [51]). Gleichzeitig ist die Möglichkeit einer gesunden Lebensweise auch eine Frage finanzieller Ressourcen.

Der Blick auf strukturellen Rassismus ist daher notwendig, um Mechanismen gesundheitlicher Ungleichheit zu verstehen und um konkrete Ansatzpunkte für nachhaltige Verhältnisprävention zu identifizieren, statt Analysen und Maßnahmen lediglich im Bereich der Beschreibungen von Gruppen und ihrer (vermeintlichen) Eigenschaften und individueller Verhaltensprävention zu verankern [29].

### Institutionen, Bildung und soziale Mobilität

Die meisten Beratungsfälle zu erlebter Diskriminierung werden in Deutschland in den Bereichen Bildung, Behörden und öffentliche Institutionen dokumentiert; diese Bereiche sind nicht ausreichend durch das Allgemeine Gleichbehandlungsgesetz geschützt [52]. Zugleich verläuft institutionelle Diskriminierung weniger sichtbar als direkte, interpersonelle und sie ist mit nachhaltigen Ausschlüssen von Personen und Personengruppen von gesellschaftlicher Teilhabe verbunden. Als Beispiel können hier die schwache soziale Durchlässigkeit und Mobilität und die geringeren Aufstiegschancen innerhalb der Bildungs- und Erwerbslandschaft Deutschlands für migrantische Personen(-gruppen) angeführt werden [53, 54]. Migrantische Schüler:innen sind signifikant geringer am Bildungserfolg beteiligt. Die Ursachen können unter anderem in der Diskriminierung an „Entscheidungsstellen“ (Schulwechsel, Fortführung der Schullaufbahn), aber auch in der fehlenden Antidiskriminierungspädagogik [55] verortet werden [15, 54]. Benachteiligung im Bildungsbereich wirkt unmittelbar auch auf gesundheitsbezogene Aspekte wie Gesundheitskompetenz, Gesundheitsverhalten, Inanspruchnahme von Versorgungs‑, Präventions- und Rehabilitationsleistungen [56].

Auch in der medizinischen Ausbildung gibt es Beispiele: So können Aspekte von bewusstem oder unbewusstem Auslassen von bestimmten Themen im Curriculum der ärztlichen Ausbildung Praktiken rassistischer oder kolonialer Wissensproduktion darstellen. So findet der Unterricht in der Dermatologie, nicht nur in Deutschland, oft nur anhand von Beispielen heller Hauttöne statt, sodass keine Diagnostik von Erkrankungen auf dunklerer Haut erlernt wird [26, 32, 57].

### Wohnsituation, Arbeit und Einkommen

Risikofaktoren für Infektionserkrankungen, aber auch für eine schlechtere psychische Gesundheit sind beengter Wohnraum und andere schlechte Unterbringungsbedingungen. Zugleich ist die Wohnsituation einer der zentralen Aspekte, die mit struktureller Ungleichheit und Benachteiligung einhergehen und von dem überproportional häufig migrantische und rassifizierte Menschen betroffen sind.

Eine Person ohne deutsche Staatsangehörigkeit hatte 2018 im Durchschnitt eine um 26 % geringere Wohnfläche zur Verfügung (21,7 m^2^ vs. 27,3 m^2^ mit deutscher Staatsangehörigkeit) und besaß seltener Wohneigentum (37 % vs. 58 %). Mieter:innen mit Migrationsgeschichte erlebten häufig Diskriminierung auf dem Wohnungsmarkt (aufgrund von Akzent, Namen, Staatsangehörigkeit oder Aufenthaltsstatus; [25]) und zahlten für vergleichbaren Wohnraum durchschnittlich mehr Miete [58]. Mit einer damit verbundenen Verdrängung aus Stadtteilen mit ansprechender Infra- und Sozialstruktur geht auch eine Deprivation im Hinblick auf Wohngegend, Anzahl der Räume, Wohnfläche oder Wohnausstattung einher [59]. In diesem Zusammenhang ist auch die Gemeinschaftsunterbringung als Risikofaktor für Infektionserkrankungen sowie psychische und allgemeine Gesundheit besonders hervorzuheben ([60, 61], s. hierzu auch den Beitrag von Führer et al. in dieser Ausgabe).

Einer besonderen Aufmerksamkeit bedarf der Themenbereich Arbeit und gesundheitliche Ungleichheit. Im Rahmen der Anwerbeabkommen für Arbeitskräfte aus einigen Ländern Südeuropas (u. a. Türkei, Italien, Griechenland, 1955–1976) wurde einer Auswahl von Arbeitskräften die Einreise und Aufnahme einer Arbeitstätigkeit ermöglicht. Die Auswahl erfolgte u. a. anhand medizinischer und gesundheitlicher Untersuchungen (Zahn- und Augengesundheit, Muskelkraft etc.; [62]). Eine Verschlechterung der gesundheitlichen Lage dieser zunächst überdurchschnittlich gesunden Bevölkerungsgruppe folgte in Verbindung mit (oft schwerer) körperlicher Arbeit, systematischen Diskriminierungs- und Ausschlusserfahrungen und Barrieren im Bereich Prävention, Versorgung und Rehabilitation [63–65].

Auch die späteren Phasen der Migration im Kontext von Flucht und dem EU-Freizügigkeitsabkommen sind durch spezifische Muster der Verschränkung von Arbeits- und Produktionsbedingungen mit der gesundheitlichen Lage von Migrant:innen und rassifizierten Personen gekennzeichnet. Sie arbeiten häufiger in zeitlich befristeten [66], informellen und ungeschützten Beschäftigungsverhältnissen [67]. Damit verbunden sind erhöhte Gesundheitsrisiken im Beruf (u. a. in „systemrelevanten Berufen“ in ÖPNV, Straßenbau, Pflege, Reinigung), etwa durch eine höhere Exposition gegenüber Infektionen, aber auch bezogen auf berufsbedingte nichtübertragbare Erkrankungen wie Verletzungen, Gelenk‑, Knochen- oder Lungenleiden [65].

Für bestimmte Gruppen von Migrant:innen gilt ein Arbeitsverbot, z. B. für Asylsuchende in den ersten 18 Monaten des Aufenthalts in Deutschland und für Personen mit prekärem Aufenthaltsstatus, was zu einem Eintritt in den informellen Sektor und illegalisierter Beschäftigung führen kann. Bei Personen ohne festen Aufenthaltsstatus kommt teils ein unklarer oder fehlender Versicherungsstatus (Kranken- und Sozialversicherung) hinzu, womit eine finanzielle und teils auch rechtliche Abhängigkeit von den Arbeitgeber:innen entsteht. Dies führt zu verstärkter Beschäftigung unter prekären und gesundheitsgefährdenden Arbeitsbedingungen bei ohnehin geminderten Arbeits- und Gesundheitsschutzregelungen.^4^

Einkommens(un)sicherheit zeigt sich ebenfalls als gesundheitsrelevant. Das Armutsrisiko von Personen mit eigener Migrationserfahrung lag 2021 deutlich höher als das von Personen ohne Migrationserfahrung (30 % vs. 23,8 %); bei Berücksichtigung des Migrationshintergrundes nach Statistischem Bundesamt (Destatis) ist die Differenz mehr als doppelt so hoch [72]. Auch hatten 48 % der Menschen mit ausländischer Staatsangehörigkeit Angst vor Arbeitsplatzverlust (42 % der deutschen Arbeitnehmer:innen; [73]). Zugewanderte waren 2021 4‑mal so stark von Arbeitsplatzverlust betroffen wie im Inland geborene Personen [74]. Zum Beispiel zeigten Arbeitsplatz- und Einkommenseinbußen in der Pandemie negative Assoziationen mit der Lebenszufriedenheit von Menschen mit ausgewählten Staatsangehörigkeiten [75].

### Rassismus und Diskriminierung im Gesundheitswesen

Rassistische Diskriminierung im Gesundheits- und Pflegesektor hat einen Einfluss auf die Versorgungssituation sowie die Inanspruchnahme von Gesundheitsleistungen. In den meisten Ländern Europas haben Migrant:innen im Gesundheitswesen ein ca. 5‑mal höheres Risiko für Diskriminierung als andere Personen [76, 77]. Informationsmangel, Sprachbarrieren, Missverständnisse oder Nicht-Verstehen und Stereotypisierungen seitens des Personals werden als Zugangsbarrieren im Gesundheitswesen kritisch diskutiert. So berichten Patient:innen von Misstrauen oder Nicht-Ernstnehmen seitens des Fachpersonals, was zu Fehldiagnosen und falschen Therapien führen kann [4, 78, 79]. Patient:innen vermeiden nach Diskriminierungserfahrungen häufiger den notwendigen Kontakt zu Ärzt:innen oder wechseln diese oft („doctor hopping“), sodass notwendige medizinische Leistungen verspätet oder gar nicht in Anspruch genommen, Behandlungsschemata nicht eingehalten oder Behandlungen abgebrochen werden [80, 81]. Dies kann eine Verschiebung von primärer zu tertiärer Versorgung, die Entwicklung von Multimorbiditäten und die Chronifizierung von Erkrankungen zur Folge haben [4, 82, 83].

### Psychische Gesundheit im Zusammenhang mit Diskriminierung und Rassismus

Der Zusammenhang zwischen Diskriminierung und psychischer Gesundheit ist deutlich [3, 4, 50, 84]. Stress, Symptomatik von Depressionen und Angststörungen, sozialer Rückzug, aber auch eine erhöhte psychische Verletzbarkeit sowie Rückgang protektiver, gesundheitsfördernder Ressourcen stehen im Zusammenhang mit erlebter Diskriminierung [4, 50].

Einige Untersuchungen weisen auf den Zusammenhang zwischen dem Gesundheitszustand der Betroffenen und den Strategien im Umgang mit erlebter Diskriminierung hin. Psychische Beschwerden zeigen höhere Werte bei Internalisierung und passivem Umgang mit Diskriminierung. Beispiele für den passiven Umgang mit Diskriminierung können Ignorieren, Kleinreden („minimizing“) oder passives Akzeptieren der negativen Erfahrung sein. Ein aktiver Umgang, wie beispielsweise das Ansprechen, Widersprechen oder die aktive Suche nach Unterstützung, wird hingegen als protektive Faktoren beschrieben [85, 86].

### Physische Gesundheit im Zusammenhang mit Diskriminierung und Rassismus

Zahlreiche Studien beschreiben Zusammenhänge zwischen Diskriminierung und physischer Gesundheit [3, 4, 87]. Auch Studien aus Deutschland zeigen z. B. Zusammenhänge zwischen Diskriminierung und der subjektiven Gesundheit [50] sowie zwischen Diskriminierung und dem Vorliegen chronischer Erkrankungen, wie etwa koronarer Herzkrankheit oder Diabetes bei Menschen mit ausgewählten Staatsangehörigkeiten (s. Beitrag Bartig et al. in dieser Ausgabe). Die allostatische Last (langfristige Abnutzungserscheinungen des Organismus, die durch chronische Stressexposition verursacht werden) fällt bei Personen, die von Diskriminierung betroffen sind, deutlich höher aus [88]. Lebenslange Konsequenzen wie Herz-Kreislauf-Erkrankungen, Bluthochdruck, Gefäßerkrankungen werden berichtet, verursacht durch erhöhte Levels von Stresshormonen wie Kortisol und weiterer Glukokortikoide sowie Katecholamine. Damit verbunden sind Stoffwechselstörungen und erhöhte Mengen von Viszeralfett [3–5, 51, 89, 90]. Erhöhte Kortisol- und Entzündungsmarkerwerte führen zu vermehrten entzündlichen und allergischen Prozessen, Betroffene können verstärkt zu rheumatoider Arthritis oder Fibromyalgie neigen. Auch wird ein Zusammenhang beschrieben zwischen Diskriminierungserfahrungen und der Verkürzung der Telomere in Zellen sowie epigenetischen Veränderungen in den Zellen der Betroffenen ([84, 91–93]; s. Exkurs in Infobox).

#### Infobox Exkurs: Biologische und physiologische Zusammenhänge von Diskriminierung und Gesundheit

Hilfreich und wichtig für das Verständnis des Zusammenhangs von Diskriminierung, Rassismus und Gesundheit ist die Analyse der Wechselwirkungen biologischer, physiologischer und sozialer Prozesse. Im Rahmen der *„ecosocial theory“* finden sich Ansätze zur Analyse von Embodiment (dt. etwa „Verkörperung“), also von Prozessen der Einschreibung sozialer Ungleichheit in den Körper, die Zellen sowie biologische und physiologische Vorgänge von betroffenen Personen [112].

Beispielsweise wurden Veränderungen der zellulären Funktionen bei Menschen mit Trauma- oder Diskriminierungserfahrungen nachgewiesen. So zeigen damit verbundene erhöhte Stressreaktionen und chronische Stressbelastungen direkte Effekte auf physiologische und zelluläre Prozesse sowie dauerhafte Veränderungen in der Genexpression und epigenetische Modifikationen [113]. Diese führen zum einen zu Veränderungen der Funktionalität des Immunsystems, u. a. zur Aktivierung einer Reihe von inflammatorischen Genexpressionsprogrammen, wohingegen die Expression von spezifischen antiviralen Immunantworten des angeborenen Immunsystems einschließlich der Immunglobulin-G-Produktion reprimiert wird, was zu einer erhöhten Suszeptibilität für virale Erkrankungen führt [113].

Zum anderen kann der mit Diskriminierung assoziierte chronische Stress (einschließlich einer kontinuierlichen niedrigschwelligen Aktivierung des Sympathikus) zu Stoffwechselstörungen, Zellerneuerungsstörungen oder vorzeitiger Zellalterung führen. Vermittelt wird dies durch erhöhte Stresshormonspiegel und -ausschüttung (u. a. Kortisol), durch Störungen im Zytokinhaushalt, Chronifizierung von Entzündungsprozessen, Störungen der Zellteilung und Zellkommunikation [114]. Es konnte gezeigt werden, dass diese Veränderungen über Generationen hinweg vererbt werden können und somit auch Auswirkungen auf die Gesundheit der Nachkommen haben können [92, 115, 116].

## Ausblick und Ansätze: Diskriminierung und Rassismus untersuchen, verstehen und adressieren

Zu den wichtigen Ansätzen gegen Diskriminierung und Rassismus im Alltag, im Gesundheitswesen und für eine diversitätssensible Weiterentwicklung der Strukturen und Prozesse in Versorgung und Forschung gehören neben dem unmittelbaren Adressieren sozialer Ungleichheit und struktureller Benachteiligung auch Antirassismus-Trainings und Aufklärungsarbeit in Institutionen wie dem Öffentlichen Gesundheitsdienst und Forschungseinrichtungen. Zugleich ist der Ausbau der Melde- und Beratungsstrukturen für Opfer rassistischer Diskriminierung und Gewalt wichtig sowie die Stärkung der Selbstorganisation und das Empowerment der Menschen mit Rassismus- und Ausschlusserfahrungen. Weitere Möglichkeiten der gesellschaftlichen Mitgestaltung und gemeinsamer Erarbeitung von rassismussensiblen Versorgungs- und Forschungskonzepten sollten geschaffen werden. Eine zentrale Grundlage ist eine offene, produktive Debattenkultur über Diskriminierung und Rassismus innerhalb der Forschungslandschaft und im Gesundheitswesen gegenüber Patient:innen, aber auch gegenüber dem Personal [94, 95]. Eine Aufklärung im Rahmen des medizinischen Curriculums über mögliche Fehldiagnostik, Leerstellen in den Lehrplänen und Diskriminierung von Patient:innen in unterschiedlichen sozialen Lagen (z. B. Versicherungsstatus, Einkommen, Herkunft) durch behandelndes Personal ist auch für die hiesigen Ausbildungsprogramme eine hilfreiche Empfehlung [96].

Auch communitybasierte Ansätze zur Gesundheitsversorgung, die auf Grundlage eines weitergefassten Gesundheitsbegriffs umgesetzt werden (der nicht nur die allgemeine Gesundheit, sondern auch die Lebensbedingungen in den Blick nimmt), sind für eine diskriminierungssensible und vielfaltsorientierte Versorgung wichtig. Als wegweisendes Beispiel sind hier Polikliniken zu nennen, die neben medizinischen auch (psycho)soziale Angebote integrieren und diese unter Einbindung der Communitys vor Ort weiterentwickeln [97, 98].

In der Forschung werden bereits neue und weiterführende Ansätze diskutiert, die zum Verständnis der Wirkmechanismen beitragen sollen. Eine Analyse der Verschränkung von Lebens‑, Wohn- und Arbeitsbedingungen und der gesundheitlichen Lage von Menschen mit Migrationsgeschichte sollte generell und in Verbindung mit Rassismus und Diskriminierung noch näher untersucht werden. Neben dem Abfragen der selbstberichteten Erfahrungen [99] sind daher auch Ansätze wichtig, die eine Erfassung von objektivierbaren Indikatoren und die Erhebung von Antidiskriminierungs- und Gleichstellungsdaten (*„equality data“*) ermöglichen können, z. B. administrative Daten oder Daten von Diskriminierungsberatungsstellen [44, 100–103] oder auch Indikatoren zur Arbeits- und Wohnsituation oder zum Zugang zu Gesundheitsversorgung [73, 104].

Bislang existieren nur wenige Studien, die eine differenzierte Analyse anhand der Indikatoren wie Einkommenssicherheit, Gesundheitsschutz oder Prekarität in Verbindung mit gesundheitlichen Outcomes zulassen. Insbesondere gilt es, auch methodisch-analytische Zugangswege zu finden, um den Anteil der erklärten Ungleichheit über verschiedene soziale Determinanten zu quantifizieren, ohne Diskriminierung und strukturellen bzw. institutionellen Rassismus als eine „pauschale“ oder „omnikausale“ Determinante zu konzipieren und zugleich ihre Wirkungsweisen innerhalb bestehender gesellschaftlicher Mechanismen gesundheitlicher Ungerechtigkeit angemessen zu interpretieren und anzuerkennen [105].

## Fazit

Diskriminierung und Rassismus sind nicht als lediglich individuelle Erfahrungen und interpersönliche Phänomene zu untersuchen und zu interpretieren. Struktureller Rassismus als gesundheitliche Determinante muss auch in der Public-Health-Forschung in den Blick genommen werden als gesellschaftliches System von Werten, Normen und institutionellen, ökonomischen, politischen und gesetzlichen Aus- sowie Einschlüssen. Dazu gehören Arbeits- und Wohnbedingungen, Zugang zu Informationen, Versorgung und angemessener Kommunikation sowie der Einfluss von geografischer und sozialer Herkunft und von rechtlichem Status auf die Gesundheit.

Die Debatten in Deutschland und Europa zu diesem Thema sind weitestgehend jung und von Interventionen migrantischer Selbstorganisationen und Nichtregierungsorganisationen geprägt [44, 106]. Zugleich entwickelt sich auch in der Wissenschaft eine Debatte um die Notwendigkeit einer sensiblen, aber auch offensiven Auseinandersetzung mit Fragen der Erhebung von (Anti)Diskriminierungsdaten und Ethnizität [44, 107–110]; denn „wer nicht gezählt wird, zählt nicht“ [44]. Geht man einen Schritt weiter, muss es sich bei erhobenen Daten um „Gleichstellungsdaten für gleichberechtigte gesellschaftliche Teilhabe“ [108, 111] handeln. Sensibel und methodisch fundiert im Rahmen von Studien und im Gesundheitsmonitoring erhobene Daten zu Diskriminierung und Rassismus können zu mehr Sichtbarkeit und auch perspektivisch zur Reduktion von gesundheitlicher Ungleichheit beitragen.

Schließlich ist auch die Übersetzung der wissenschaftlichen Evidenz in politische Entscheidungen notwendig. Dies beinhaltet neben Forschung auch Evaluation von Maßnahmen, Ableitung von Handlungsempfehlungen und Umsetzung von konkreten Programmen unter Einbeziehung relevanter intersektoraler Partner:innen. Dazu gehören Maßnahmen im Bereich der sozialen und strukturellen Determinanten gesundheitlicher Ungleichheit, die Ermöglichung eines gleichberechtigten Zugangs zu guter Gesundheit und der Abbau von Diskriminierung, Rassismus und sozialer Ausgrenzung.

## Footnotes

1 Mit *weiß* ist nicht der Phänotyp der Haut eines Menschen gemeint, sondern eine gesellschaftspolitische Norm und Machtposition, *weiß* wird daher klein und kursiv geschrieben. Mit Weißsein ist die privilegierte und dominante Position innerhalb des Machtverhältnisses Rassismus gemeint, die sonst unausgesprochen und unbenannt bleibt. Schwarz wird stets groß geschrieben, damit wird verdeutlicht, dass es sich nicht um eine (Haut)Farbe handelt, sondern um eine Selbstbezeichnung, die die Lebensrealitäten und die gesellschaftspolitische Position von Menschen im Kontext der gemeinsamen Erfahrungen mit (Anti-Schwarzem) Rassismus beschreibt.

2 Ein Beispiel hierfür stellt das Asylbewerberleistungsgesetz (u. a. § 4, 6) dar, welches den Zugang zu Sozial- und Gesundheitsleistungen für Asylsuchende in Deutschland beschränkt. Ein anderes Beispiel ist die Pflicht nach § 87 Abs. 2 Nr. 1 Aufenthaltsgesetz für Sozial- und Versorgungseinrichtungen, Name und Aufenthaltsort von Menschen ohne gültigen Aufenthaltsstatus an die Ausländerbehörde zu übermitteln, wodurch eine Inanspruchnahme notwendiger medizinischer Versorgung erschwert bis verunmöglicht wird [35].

3 Nicht alle Menschen mit Migrationsgeschichte machen Rassismuserfahrungen im Alltag und/oder sind von strukturellem und institutionellem Rassismus betroffen. Zugleich haben rassifizierte und ethnisierte Menschen nicht zwingend eine eigene oder familiäre Migrationsgeschichte, wie beispielsweise jüdische Menschen, Schwarze Deutsche oder Sinti und Roma, deren Familien teilweise seit mehreren Hundert Jahren in Deutschland leben. Da die Datenlage zu Rassismus, Diskriminierung und damit verbundener struktureller Benachteiligung in Verbindung mit entsprechenden Indikatoren („Antidiskriminierungs- und Gleichstellungsdaten“) in Deutschland bislang jedoch mangelhaft ist, wird zur Analyse häufig und im vorliegenden Beitrag stellenweise als Proxy die eigene oder familiäre Migrationsgeschichte herangezogen [44].

4 In der COVID-19-Pandemie zeigten sich die ohnehin vorhandenen gesundheitsgefährdenden Effekte einer solchen Lage besonders deutlich: körperliche Arbeit, unzureichender Arbeitsschutz, begrenzter oder kein Zugang zu Gesundheitsversorgung und persönlicher Schutzausrüstung, das Fehlen von mehrsprachigen gesundheitsrelevanten Informationen über Rechte und Infektionsschutz, keine Möglichkeit, im Krankheitsfall Krankengeld und Sozialleistungen zu erhalten, bis hin zu „Anwesenheits‑“ oder „Erscheinungsprämien“ für Arbeit im Lockdown [68]. Diese Faktoren haben zu erhöhten Infektions- und Erkrankungsrisiken in Beschäftigungssektoren geführt, in denen überproportional häufig Migrant:innen vertreten sind [66–71]. Spezifische Risiken und zugleich mehr Unsicherheit sowie weniger Schutz stellten eine Kombination dar, die auch jenseits von Krisenlagen relevant für akute und langfristige gesundheitliche Probleme ist.
